# Distribution of ^137^Cs in benthic plants along depth profiles in the outer Puck Bay (Baltic Sea)

**DOI:** 10.1007/s10967-012-1723-0

**Published:** 2012-03-25

**Authors:** Tamara Zalewska

**Affiliations:** Institute of Meteorology and Water Management−National Research Institute, Maritime Branch, Waszyngtona 42, 81-342 Gdynia, Poland

**Keywords:** Radioactive cesium, Marine phytobenthos, Biomass influence, Baltic Sea

## Abstract

A study was conducted on three macroalgae species: *Polysiphonia fucoides* and *Furcellaria lumbricalis*, the species of the red algae division, and *Cladophora glomerata*, representing the green algae division, as well as *Zostera marina*, representing vascular plants. The main aim of the study was to recognize the level of ^137^Cs concentrations in the plants, which could be used as a measurement of bioaccumulation efficiency in the selected macrophytes at varying depths, and in the seasonal resolution of the vegetation period: spring–summer and autumnal. The plants’ biomass clearly showed seasonal variability, as did the ^137^Cs concentrations in the plants. Cesium activity also changed with depth. Seasonal variability in radionuclide content in the plants, as well as the differences in its activity determined along the depth profile, were related mainly to the plant biomass and the dilution effect caused by the biomass increment and reflected the growth dynamics. *P. fucoides* showed much greater bioaccumulation ability at each depth as compared to *C. glomerata*, a green algae. Lower concentrations of ^137^Cs were also identified in *F. lumbricalis* and in *Z. marina,* mostly as a result of differences in morphology and physiology. *P. fucoides* can be recommended as a bioindicator for the monitoring of ^137^Cs contamination due to the high efficiency of bioaccumulation and the available biomass along the depth profile, as well as the occurrence throughout the entire vegetation season.

## Introduction

Benthic plants are an inherent element of the marine environment with the characteristic feature of a considerable bioaccumulation ability of elements, both those of physiological importance as well as potentially toxic ones [1–12]. This feature is relevant mainly in the case of nutrients—indispensable for the photosynthesis and plant growth, but also in the case of heavy metals and specifically radionuclides [2].

The bioaccumulation process occurring in plants may be applied to environmental studies in many areas. Macrophytobenthic organisms are considered as potential reference organisms to be used in models for assessing the radiological consequences of radioactive releases, arising both from the routine operation of nuclear facilities [13] and from accidental situations [14]. With regard to heavy metals, which are potentially hazardous to the environment, numerous benthic plant species, characterized by highly effective bioaccumulation ability, are considered for application as cleaning agents in aqueous reservoirs [15, 16]. However, primarily, benthic plants are used as bioindicators for monitoring surveys of environmental pollutants [10, 12, 17–20].

The specific features of bioaccumulation observed in benthic plants make them useful as indicators of environmental status, both in steady state conditions when the balance between the input (sources) and the output is observed, as well as in situations of accidental pollution of the marine environment by heavy metals or radionuclides.

One such feature is the response of a given bioindicator that occurs at a relatively short time interval after a pollution event [21]. In contrast to benthic fauna or fish, which accumulate hazardous substances through their entire life span, the level of radionuclides in benthic plants results mainly from a single year of vegetation.

The concentration of elements accumulated in plants, including heavy metals and radionuclides, is not directly related to the relevant contents in the surrounding environment. Bioaccumulation is determined by external and internal factors [2, 22]; therefore the knowledge of bioaccumulation processes is of importance. The external factors are physical and chemical factors relevant to the surrounding environment. Physical factors are temperature, light conditions, and water motion, which could be characterized by currents and salinity. Physical factors all show definite seasonal variability, though salinity to a lesser extent. The chemical factors detrimental to the bioaccumulation process are the concentration of the investigated trace element and its physical and chemical form, which can facilitate or hamper the element uptake. Internal factors are biological factors related to the morphology and physiology of the investigated organisms [2, 7, 19, 20, 22–24]. It is very difficult to distinguish explicitly and determine which factor is the most influential in bioaccumulation in a chosen species, and in a defined period. This is especially true when taking into account that physical factors, e.g. temperature and irradiance, are in close correlation with physiology in influencing the intensity of vital processes and, as consequence, the plant biomass. The evidence that the magnitude of biomass was the factor determining the final concentration of a metal in the plant tissue was presented in a number of publications, e.g. it was found that in *Ulva* and *Enteromorpha*, seasonal variation in the content of different metals was caused by dilution during the period of maximum growth, and concentration during the period of slow growth [18, 25]. Similar seasonal characteristics were obtained in the case of Cu and Cd in *Ulva rigida*, in which the increase of biomass resulted in the decrease of both metals [18, 26–28]. A strong seasonality in ^137^Cs bioaccumulation was directly connected with physiological activity and hence the biomass was detected in macrophyte species *Polysiphonia fucoides* and *Zostera marina* [29].

The primary aim of the study was the determination of concentrations of the radionuclide ^137^Cs as a measure of bioaccumulation processes in selected benthic plants at different depth levels. The influence of biomass, which is a function of depth (i.e. irradiance conditions) and seasonal conditions, on ^137^Cs concentrations in plant tissue was examined. The study was conducted on three macroalgae species: *P. fucoides* and *Furcellaria lumbricalis*, the species of the red algae division and *Cladophora glomerata* representing the green algae division, and *Z. marina*, representing vascular plants. The selected species are specific to the flora of the southern Baltic Sea and can be used as potential bioindicators of pollution of the marine environment [3].

## Materials and methods

### Sampling

**Fig. 1:**
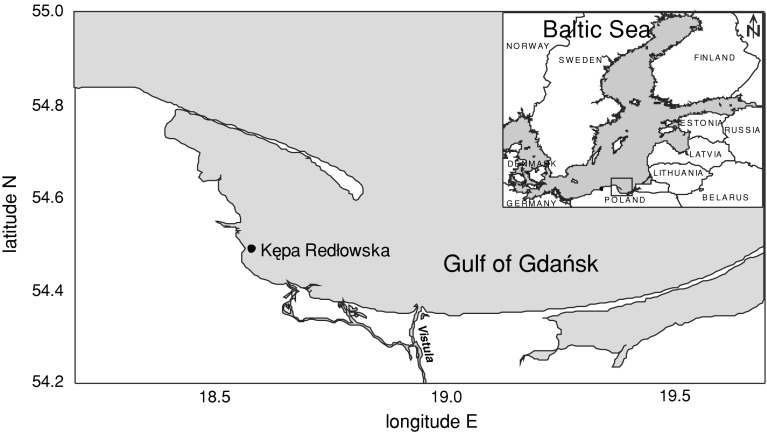
Sampling location for macrophytobenthic plants

Benthic plants were collected in the outer Puck Bay in the southern Baltic Sea, at a location called Kępa Redłowska (Fig. 1). The plants were collected along a depth profile from 1 to 7 m, at 1 m intervals, in July and September 2008, in May and October 2009, and in June and October 2010. Sampling was conducted by a scuba diver who collected the plants enclosed in a metal frame of 0.5 × 0.5 m from the sea floor. At each depth the frame was deployed 3 times and the ensuing sub-samples were integrated; the results were then related to the stretch of 0.75 m^2^ (3 × 0.25 m^2^). Integrated samples secured in plastic bags were then transported to the laboratory, where further preparation of the samples was conducted.

Data on water temperature measured in Gdynia (Fig. 1) in the period 2008–2010 were taken from the monitoring service operated by the Observational Service of the Institute of Meteorology and Water Management in Gdynia.

The data on current velocity on the sampling dates (28 June and 1 October 2010) were generated with a model MIKE operating in the Department of Oceanography and Baltic Sea Monitoring of the Institute of Meteorology and Water Management, Maritime Branch (http://baltyk.imgw.gdynia.pl/).

### Sample preparation

The plant material was analyzed taxonomically; individual species occurring in the samples were identified and the biomass of each species was determined gravimetrically; taxonomic analyses and biomass determinations were carried out according to the HELCOM COMBINE guidelines [30]. ^137^Cs concentrations were examined solely in plant species having biomass sufficient to measure the radionuclide activity with adequate accuracy. In practice, species with biomass <1 g were not included in the radioactivity analyses.

To determine the radionuclide activity, plant samples were dried, ashed at 450 °C and homogenized. Homogenized samples were placed in counting boxes of an appropriate shape and size.

### Analysis and measurement

^137^Cs content in samples of biological material was measured by the gamma spectrometry method using an HPGe detector with a relative efficiency of 18 % and a resolution of 1.8 keV for peak of 1,332 keV of ^60^Co. The detector was coupled with an 8192-channel computer analyser and GENIE 2000 software. The reliability and accuracy of the applied method was verified by participation in the inter-comparison exercises organized by IAEA-MEL Monaco (IAEA-414, Irish and North Sea Fish) [31]. Repeated analysis gave a value equal to 5.06 ± 0.64 Bq kg^−1^ d.w. while the estimated target value was equal to 5.18 ± 0.10 Bq kg^−1^ d.w.

## Results and discussion

The study was conducted on plant species typical of the southern Baltic Sea region, while the selection of radioactive ^137^Cs was prompted by the fact that it is a nuclide of solely anthropogenic origin and its activities in the marine matrices in the Baltic Sea are much higher than those of other artificial isotopes [32–36]. The increased levels of ^137^Cs are directly related to the Chernobyl nuclear plant accident in 1986, in which a large amount of this isotope was released into the Baltic Sea. It is estimated that the overall load of ^137^Cs that reached the sea from this source was 4.7 TBq, comprising 82 % of the total amount of cesium 137 which was accumulated in the Baltic seawater [33, 37]. In the case of radioactive cesium, its concentration is almost uniform in the area of interest [35]. Changes in cesium concentrations are negligible in the investigated period. It occurs mainly in the cationic form, and therefore the chemical factors, which could influence the cesium level in the plant tissue, can be excluded from the discussion.

**Fig. 2:**
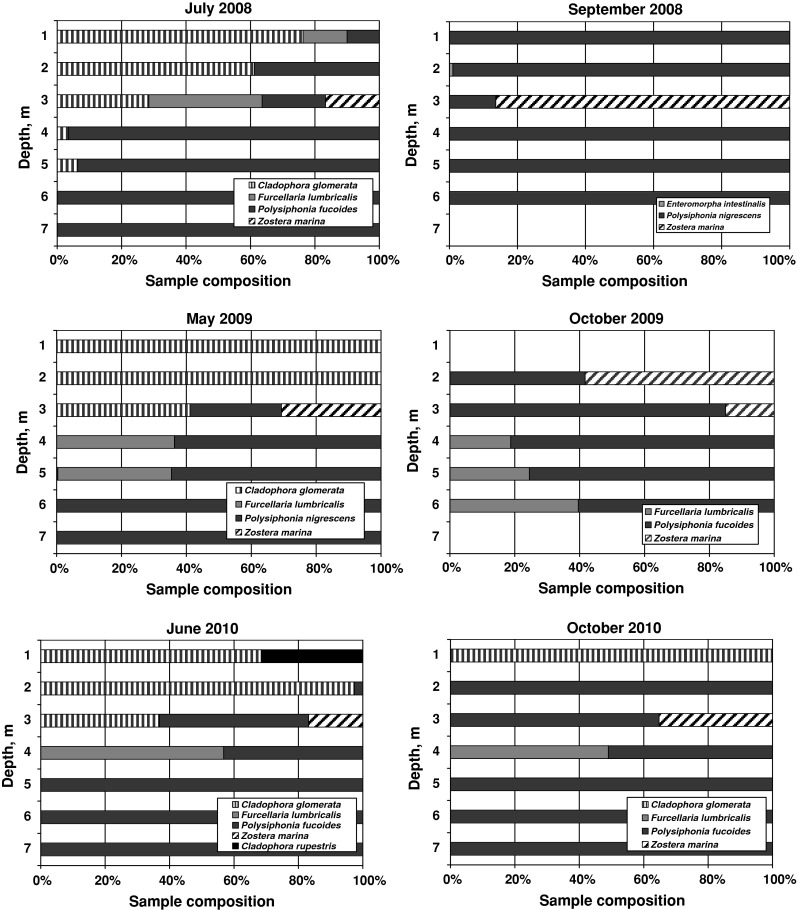
Taxonomic composition of samples taken in July and September 2008, in May and October 2009 and in June and October 2010

Phytobenthos community structure, as well as species occurrence at particular depth intervals, depended on the sampling period (Fig. 2). Sampling along the depth profile in July 2008, May 2009 and June 2010 revealed the predominance of *C. glomerata*, a representative of green algae, at the depth of 1–3 m. Additionally, a *Cladophora* genera—*Cladophora rupestris*—was identified in June 2010 at 1 m depth. On all sampling occasions the sea grass, *Z. marina*, was found at the depth of 3 m, this vascular plant having, similarly to *C. glomerata*, chlorophyll *a* as the sole assimilation pigment. The wavelength of light, indispensable to photosynthetic reactions with particular assimilation pigments, is the main factor in determining the depth limit of plants. In July 2008, a few shoots of *C. glomerata* were also identified at 4 and 5 m depths, this probably being a result of irradiance and water clarity conditions at these depths. Every year, in spring–summer time, the domination of species changed to *P. fucoides* and *F. lumbricalis* at greater depth. Both species represent the red algae division and contain phycobilins as well as chlorophyll *a*. In autumn, *P. fucoides* dominated along the entire depth profile (Fig. 2), except for the section at 3 m depth in 2008 and at 2 m depth in 2009, where *Z. marina* prevailed in terms of biomass. On the latter sampling occasion, the absence of green algae, the shift of the main *Z. marina* biomass to a shallower area (from 3 m depth to 2 m in 2009) and the absence of benthic plants at 7 m depth were the direct results of deterioration in living conditions, i.e. decline in light conditions and temperature decrease. However, the presence of *C. glomerata* at 1 m depth in October 2010 was a highly unexpected observation, the more so, that its biomass was comparable with that recorded in June this year. It was probably the result of exceptionally warm (13.3 °C) and sunny weather on 1 October 2010.

**Fig. 3:**
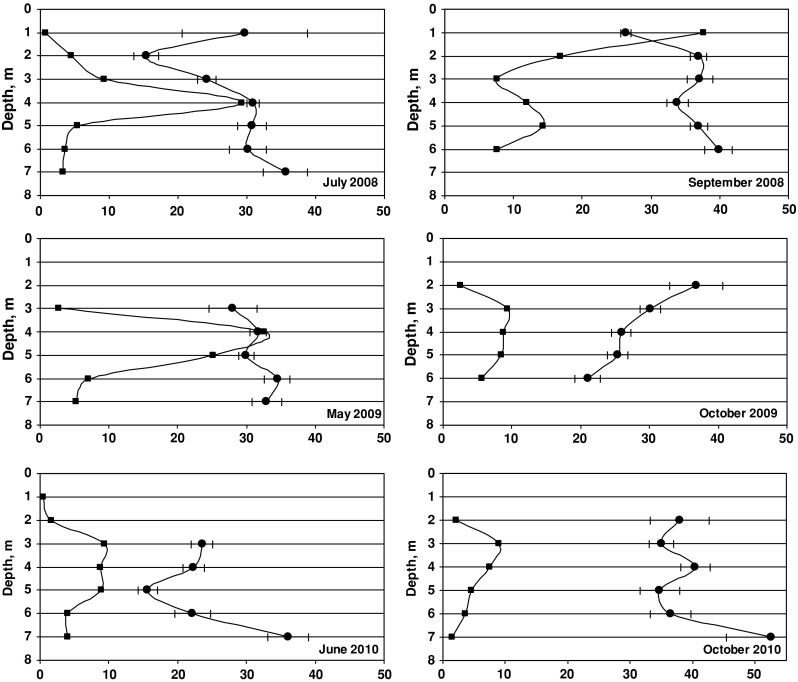
Changes of ^137^Cs activity concentrations (*filled circles*) and biomass (*filled squares*) of *P. fucoides* as a function of depth (*errors bars* represent counting errors—1σ)

**Fig. 4:**
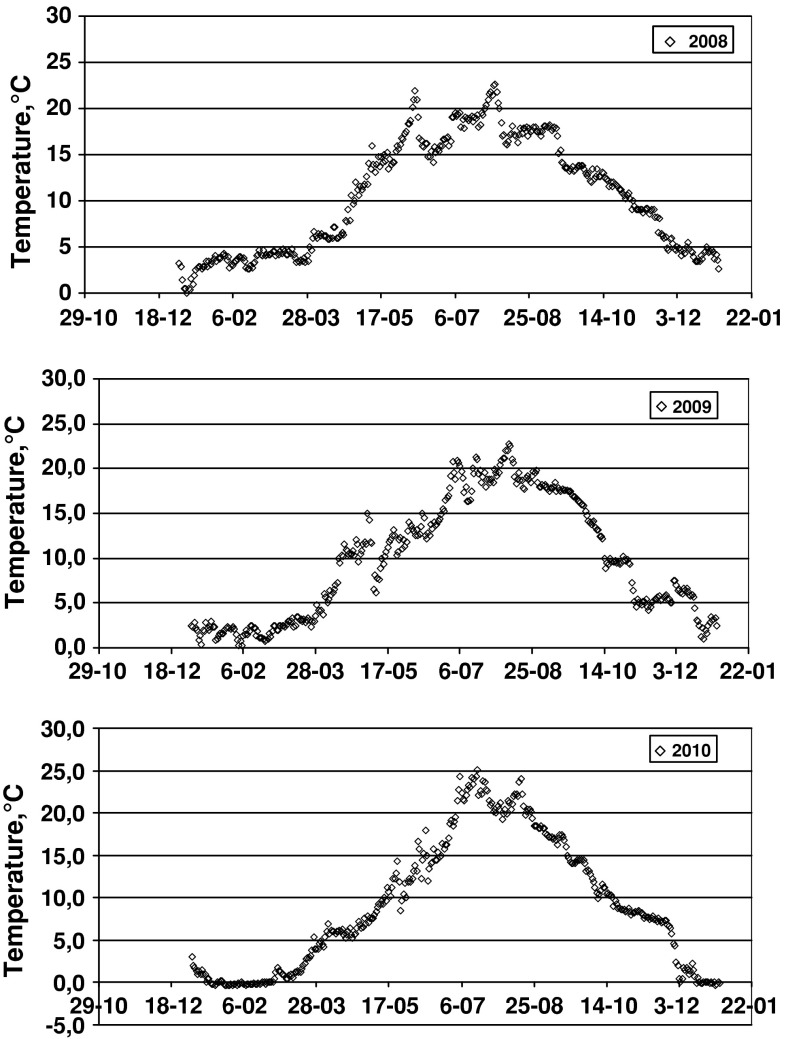
Seawater temperature in Gdynia in the years 2008–2010

Consequently, the biomass of particular species changed accordingly with the sampling period and sampling depth. Sampling from a well-defined bottom area (0.75 m^2^) enabled the comparison of species biomass at various depths and during various stages of the vegetation season. Changes in ^137^Cs activity concentration in *P. fucoides* versus the plant biomass in samples along the depth profile are presented in Fig. 3. The biomass curves from July 2008 and May 2009 have very similar shapes within the depth range of 3–7 m; a significant increase in plant biomass was observed between 3 and 4 m of depth, the curve reaching its maximum at the deeper level. This may be explained by the light conditions, which seemed to be optimal for the assimilation processes of red algae in the 3–5 m depth range. The effect of temperature can be dismissed, as the differences along such a shallow water profile are either very small or nonexistent [29]. Additionally, a comparable magnitude of plant biomass in July 2008 and in May 2009, when the thermic conditions differed considerably (seawater temperature was 23.3 °C in July and 8 °C in May), might be indicative of a much lesser influence of temperature on biomass increments (Fig. 4). In June 2010, an important difference in biomass development of *P. fucoides* along the depth profile was observed—a much smaller biomass was collected between 3 and 5 m. This observation supported the hypothesis of a limited effect of temperature on *P. fucoides* biomass growth, as its biomass was nearly 3 times bigger at 8 °C on 8 May 2009 than at 16.3 °C on 28 June 2010 (Figs. 3, 4). The reduced biomass of *P. fucoides* observed in June 2010 could be the result of a change in environmental conditions in the Gulf of Gdańsk due to the flood event that occurred that year. The first flood crest of the Vistula river flood discharged into the Gulf of Gdańsk on ca*.* 24 May 2010 and the second on 12 June 2010. The floodwater contained large amounts of nutrients and suspended matter, hence water transparency in the Gulf as well as oxygen conditions in near-bottom areas decreased considerably. These two factors were presumably responsible for the reduction of *P. fucoides* biomass.

In July 2008, ^137^Cs concentrations in plants varied in a relatively wide range from 15.4 to 35.6 Bq kg^−1^ d.w. The remarkable increase in ^137^Cs concentration in plant tissue observed between 3 and 5 m depths can be related to enhanced metal uptake being a consequence of an increase in metabolic activity at the optimal depth range. On the other hand, the increase in biomass causes a so-called diluting effect, decreasing radionuclide concentration; hence the final isotope content in the plant can be seen as a result of these opposite processes. Zalewska [29] has indicated that the final concentration of ^137^Cs in a plant is the result of multiple processes whose development is determined by the plant growth phase, or the intensity of living processes, and these are directly related to environmental factors.

^137^Cs concentrations in *P. fucoides* at 4–6 m depth in July 2008 and May 2009 were on the order of 30 Bq kg^−1^ d.w., while those measured in June 2010 reached ca*.* 10 Bq kg^−1^ d.w. This difference is indicative of the fact that the dominating factor affecting the bioaccumulation of radioactive cesium is the intensity of living processes, specifically the magnitude of biomass in this case, which was much bigger in 2008 and 2009.

The most interesting feature presented in Fig. 3 is the correlation between the decreasing biomass of *P. fucoides* and the increasing ^137^Cs concentration observed in the deepest parts of the profiles in July and September 2008, and especially marked in June and October 2010. A similar relationship was observed in the very shallow part of the profile (1 m depth) where a very small biomass of *P. fucoides*, identified in July 2008 and October 2009, was discovered to contain large concentrations of ^137^Cs. One of the possible interpretations of the ^137^Cs distribution correlation with biomass relates to the fact that in an aqueous environment, a certain reservoir of Cs^+^ ions appears at each depth and its bioaccumulation can be limited by the rate of diffusion to the laminar water layer adherent to the plant surface. The external surface of algae plays a very important role in the bioaccumulation process; as a consequence of intensive accumulation across a large exchange surface, the concentration of ^137^Cs in the water might decrease considerably in direct proximity to the plant, causing an inhibition of the process. At depths where the biomass increase was lower, the transport of Cs^+^ ions to the laminar water layer was less inhibited.

**Fig. 5:**
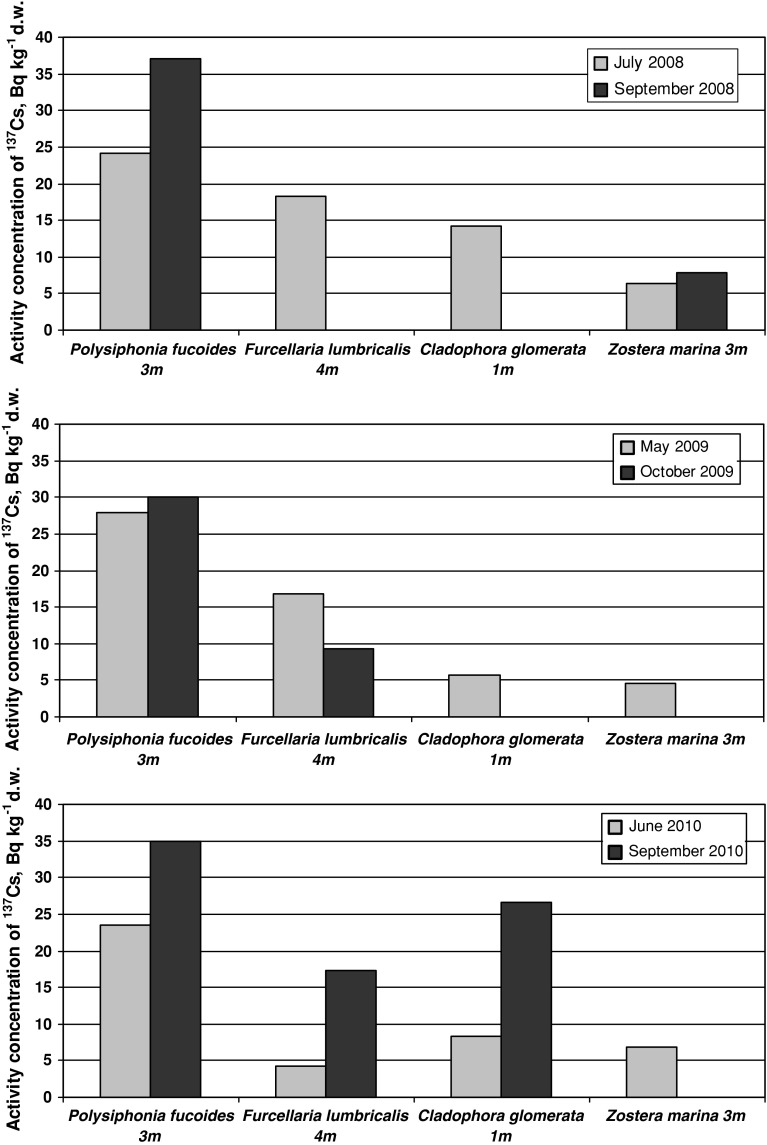
Comparison of ^137^Cs activity concentrations in *P. fucoides*, *F. lumbricalis*, *C. glomerata* and in *Z. marina* found in spring–summer and autumn

The curves of biomass and ^137^Cs activity distribution against depth were notably different in autumn after light conditions had deteriorated and temperature decreased. In September 2008 and in October 2009, a significant decrease in *P. fucoides* biomass was observed regarding the optimal depth interval. Vertical distribution of *P. fucoides* biomass in October 2010 was similar to that recorded in October 2009 and June 2010, as described above. At the same time, ^137^Cs activities measured in autumn (in the entire period of study) were definitely higher than the ones measured in spring–summer at almost all depth levels. Additionally, regarding the particular plant species at selected depth intervals, higher activities of ^137^Cs were observed in *P. fucoides* in autumn in all years of the study (Fig. 5), in *Z. marina* in 2008 and in *F. lumbricalis* and *C. glomerata* in 2010. It has to be noted that in 2010, the differences observed between summer and autumnal ^137^Cs concentrations were maximal. The explanation of this observation can be made taking into account three aspects.

The first one was discussed in detail in Zalewska [29] where it was explained that in summer, under optimal growth conditions, intensive plant growth and fruiting result in great increases of biomass. These growth processes are directly related to metabolic processes: photosynthesis and respiration. Simultaneously, as the intensification of metabolic processes results in enhanced metal uptake, the augmentation of biomass causes a diluting effect. Therefore, the final concentrations of cesium in the plants in summer are resultant of these two processes. In autumn, as in summer, there are two opposing tendencies that affect cesium content in the plant tissues. Metabolic processes are still rather intensive and enhance bioaccumulation but, on the other hand, the decline in thermal and insolation conditions act against the metabolic activity and slow it down. This slowing down causes a decrease in the rate of bioaccumulation and is also seen in biomass decline, although the latter effect can be considered as a concentration process (opposite to the diluting process connected to the increment of biomass). The final effect, resulting from these opposing activities, is the continued increase of ^137^Cs concentration in plant tissue in autumn.

The second possible explanation of the increase of ^137^Cs concentration in plant tissues can be the fact that the dying plants (this situation being much more intensive in autumn) can release cesium directly from the tissue into the seawater, in this way supplying additional cesium loading to the environment.

**Fig. 6:**
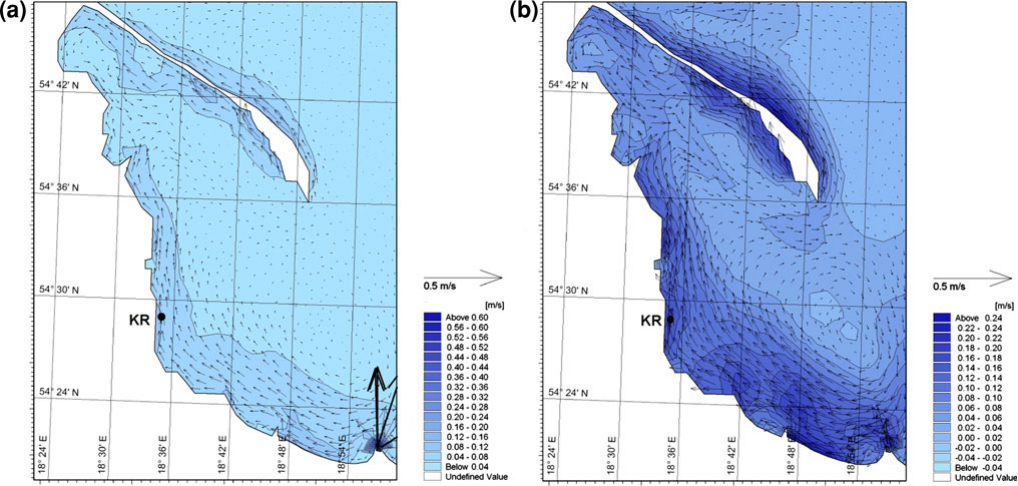
Currents speed in Outer Puck Bay in June (**a**) and October (**b**) 2010

Still another factor that can influence the ^137^Cs concentrations observed in plants during the autumn might be connected with hydrological conditions, with special attention paid to water motion expressed as the speed of sea currents. Water motion is important regarding the transport of ions to the surface of the thallus [2]. When the speed of marine currents is low the transport of ions, and simultaneously bioaccumulation, might be more limited by diffusion (as mentioned earlier). In autumn, the speed of currents in the region of investigation could be one order of magnitude higher then that observed in summer (Fig. 6).

**Fig. 7:**
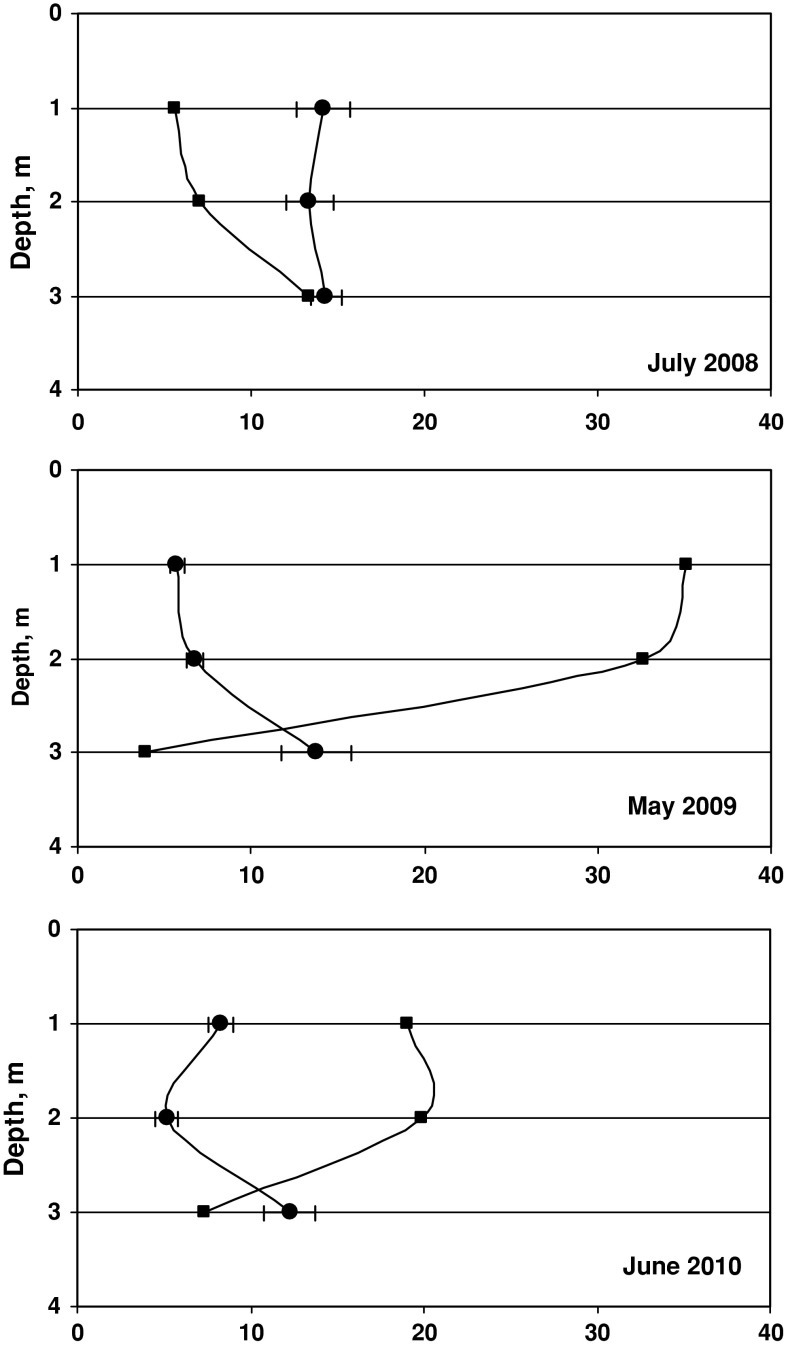
Changes of ^137^Cs activity concentrations (*filled circles*) and biomass (*filled squares*) of *C. glomerata* as a function of depth (*errors bars* represent counting errors—1σ)

The curves of biomass and ^137^Cs distribution, as a function of depth, were also examined in the case of *C. glomerata* (Fig. 7). This species occurred in a narrow depth range of 1–3 m, determined by the availability of light, which is indispensable in the assimilation processes of chlorophytes. In May 2009, the largest algae biomass was determined at the 1 m depth, and this was accompanied by a minimal level of ^137^Cs activity, ca*.* 5 Bq kg^−1^dw. In June 2010, the general features of ^137^Cs concentrations and biomass profiles were similar to those observed in the previous year, except that the maximal biomass of *C. glomerata* was markedly smaller. Simultaneously, as in the case of *P. fucoides* this fact cannot be explained by thermic conditions. The concentration of ^137^Cs increased with the decrease in *C. glomerata* biomass, as in the case of *P. fucoides*. In July, the differences in biomass along the depth profile were much smaller and the concentration of ^137^Cs remained at a relatively stable level of ca. 14 Bq kg^−1^ dw.

The comparison of ^137^Cs concentrations in *P. fucoides* and *C. glomerata* showed much higher values in the red algae than in the chlorophyte species. These two algae belong to the filamentous group, one of the groups in the functional-form model proposed by Littler [2, 38]. Littler’s model assumes that functional characteristics (e.g. uptake of environmental elements) depends on the morphology and surface to volume ratios. *P. fucoides* and *C. glomerata* are both characterized by a very large external surface exchange area, the most important morphological parameter, which remains in close relation to the method of exchange. Macroalgae exchange elements with the environment by foliar uptake and therefore, the exchange surface is crucial to the bioaccumulation process. Lower concentrations of ^137^Cs, as compared to *P. fucoides*, were observed also in *F. lumbricalis*, a red algae of different tissue build, which influences the effectiveness of bioaccumulations as stipulated by Zalewska and Saniewski [19, 20]. The other internal factor, besides morphology, which determines bioaccumulative processes, is the physiology of the investigated organisms. Lower concentrations of ^137^Cs were also observed in vascular plant *Z. marina* (Fig. 6), which exchanges elements with the environment mainly via a root system, what might be crucial for bioaccumulation and for final isotope concentration.

## Conclusions

^137^Cs accumulation by benthic plants was examined in the shallow coastal area of the outer Puck Bay at a specific depth profile and in seasonal resolution. Species structures and their biomass, as well as radionuclide concentrations in plants, showed clear seasonal variability.

Seasonal changes in ^137^Cs concentrations reflect the growth dynamic of the plants and are attributed to the diluting effect in periods of intensive biomass increment. An increase in ^137^Cs content observed in autumn might be related to the availability of additional radionuclide amounts released directly from the dying plant tissues and also by the facilitated transport of ions due to the increased speed of currents.

*P. fucoides*, a red algae, showed much greater bioaccumulation ability at each depth when compared to *C. glomerata*, a green algae, despite the fact that both species are characterised by large exchange surfaces, a feature which is highly influential in the bioaccumulation process. Lower concentrations of ^137^Cs were also identified in *F. lumbricalis,* belonging to the red algae division, and in *Z. marina,* representing vascular plants. In all cases the biological factors, physiology and morphology, were found to be responsible for the final bioaccumulation efficiency. *P. fucoides* can be recommended as a bioindicator for the monitoring of ^137^Cs contamination due to the high efficiency of bioaccumulation and the available biomass along the depth profile, and the occurrence throughout the entire vegetation season.
